# Small Molecule Induced FLT3 Degradation

**DOI:** 10.3390/ph15030320

**Published:** 2022-03-08

**Authors:** Sun-Young Han

**Affiliations:** College of Pharmacy and Research Institute of Pharmaceutical Sciences, Gyeongsang National University, 501 Jinju-daero, Jinju-si 52828, Korea; syhan@gnu.ac.kr

**Keywords:** fms-like tyrosine kinase 3, target protein degrader, heat shock protein 90, ubiquitin proteasome system

## Abstract

Target protein degrader is a new paradigm in the small molecule drug discovery field and relates to the term ‘event-driven pharmacology’. Fms-like tyrosine kinase 3 (FLT3) is a significant target for treating acute myeloid leukemia (AML). A few FLT3 kinase inhibitors are currently used in the clinic for AML patients. However, resistance to current FLT3 inhibitors has emerged, and strategies to overcome this resistance are required. Small molecules downregulating FLT3 protein level are reported, exhibiting antileukemic effects against AML cell lines. Small molecules with various mechanisms such as Hsp90 inhibition, proteasome inhibition, RET inhibition, and USP10 inhibition are explained. In addition, reports of FLT3 as a client of Hsp90, current knowledge of the ubiquitin proteasome system for FLT3 degradation, the relationship with FLT3 phosphorylation status and susceptibility of FLT3 degradation are discussed.

## 1. Introduction

The most crucial trend in the small molecule drug discovery area is the employment of the target protein degrader concept, identifying compounds that induce the removal of the target protein. Most small molecule drugs currently used in the clinic are target inhibitors modulating target protein function. There are terms for these two drug discovery paradigms: event-driven pharmacology and occupancy-driven pharmacology [1]. Event-driven pharmacology indicates the target degradation, while occupancy-driven pharmacology usually means inhibition of the target by drug-binding. The introduction of event-driven pharmacology expanded the pool of “druggable’ proteins. Some “undruggable” proteins, as in terms of occupancy-driven pharmacology due to the lack of drug binding sites for protein function modulation, became “druggable” as a result of event-driven pharmacology. The advent of proteolysis-targeting chimeras (PROTAC) technology as a drug discovery platform also accelerated the development of target protein degraders. A detailed description of PROTAC can be found in several review papers [1,2].

AML is a hematologic malignancy characterized by the proliferation of immature myeloid cells [3]. The five-year survival for AML patients is approximately 30%, indicating poor outcomes for the AML patients [4]. Standard intensive chemotherapy called the 7+3 regimen, a combination of seven days of cytarabine and three days of anthracycline, has been used for more than 40 years [4]. Less intensive therapy such as hypomethylating agents (azacitidine and decitabine) is available for older patients who cannot tolerate intensive treatment. Recently several targeted therapies for better efficacy and less toxicity were developed. Venetoclax, with a mechanism B cell lymphoma-2 (BCL-2) inhibition, ivosidenib and enasidenib inhibiting isocitrate dehydrogenase activity, glasdegib that inhibits the hedgehog pathway, and Fms-like tyrosine kinase 3 (FLT3) inhibitors are new agents for the treatment of a subset of AML [3].

FLT3 is receptor tyrosine kinase expressed on hematopoietic cells as well as acute myeloid leukemia (AML) cells, regulating cell survival and proliferation [5]. FLT3 is one of the major targets for AML therapy, and activating mutations of FLT3 are found in approximately one-third of AML and less than 3% of acute lymphoblastic leukemia patients [6,7]. Internal tandem duplication of FLT3 (FLT3-ITD) within the juxtamembrane domain and point mutation in kinase domain (e.g., D835Y) are representative mutations present in AML patients, resulting in constitutive activation of kinase activity [6,8]. Small molecules inhibiting the kinase activity of FLT3 have been developed, and two FLT3 inhibitors, midostaurin and gilteritinib, are currently used in clinics for AML patients [9,10].

Similar to other kinase inhibitors [11], a challenge for FLT3 inhibitors is also the emergence of acquired resistance [12]. Resistance can be divided into on-target and off-target effects. Off-target resistance is mediated by several mechanisms other than the modification of FLT3. Identified off-target mutations in patients with FLT3 inhibitor treatment include alteration of downstream signaling pathways, epigenetic regulators, and myeloid transcription factors [13]. On-target resistance occurs by secondary mutations of the FLT3 gene. Two major mutations are D835Y and F691L mutations located in the tyrosine kinase domain [14]. D835Y, a mutation in the activation loop, confers resistance by promoting the active conformation of FLT3 [15]. Apart from the D835Y mutation found as a cause of AML, FLT3 D835Y as a secondary mutation sometimes results in the dual mutation of FLT3-ITD/D835Y. FLT3/F691L mutations in the gatekeeper residue induces a lower binding affinity to FLT3 inhibitors [16]. Next-generation FLT3 inhibitors are needed to overcome the resistance against current FLT3 inhibitors [17]. 

While most investigations have concentrated on the kinase activity inhibition of FLT3 for AML therapeutics, several small molecules downregulating the FLT3 protein expression have also been reported. These FLT3 downregulators also exhibited the anti-proliferative effect on AML cell lines harboring FLT3 mutation. These reports demonstrate that FLT3 degrader, as well as FLT3 activity inhibitor, can be an effective therapeutic strategy for AML patients. The research regarding the small molecules with the activity of downregulating FLT3 protein level and the mechanism will be explained in this review.

## 2. HSP90-Mediated FLT3 Degradation

### 2.1. Hsp90 Inhibitors

Heat shock protein 90 (hsp90) is a molecular chaperone that stabilizes its client proteins by forming multiprotein complexes. Inhibition of Hsp90 function can dissociate this multiprotein complex and result in the degradation of client proteins. Small molecule-induced FLT3 degradation is first reported in Hsp90 inhibitor treatment. Treatment of Hsp90 inhibitors such as 17-allylamino-17-demethoxy-geldanamycin (17-AAG) resulted in the reduced expression of FLT3, suggesting FLT3 degradation by dissociation from its chaperone [18]. In leukemic cell lines expressing FLT3 wild type or FLT3-ITD, 17-AAG treatment resulted in a significant decrease in FLT3 protein. This observation led to the discovery of FLT3 as an Hsp90 client protein, as discussed below.

### 2.2. Green Tea Catechins

The next small molecules inducing FLT3 degradation to be reported are green tea catechins [19]. Green tea polyphenols were subjected to AML cell proliferation assay and FLT3 Western blot. The (-)-epigallocatechin-3-gallate (EGCG), (-)-epigallocatechin (EGC), and (-)-epicatechin-3-gallate (ECG) suppressed AML cell proliferation. These three catechins also downregulated FLT3 expression in a variety of AML cell lines. The mechanism of FLT3 downregulation was pursued, and the Hsp90 protein appeared to mediate the FLT3 downregulation. Upon EGCG, EGC, and ECG treatment, the association of Hsp90 with client protein FLT3 was disrupted, as shown by immunoprecipitation. Interestingly, FLT3 downregulation was only observed in AML cell lines with FLT3 mutation (FLT3-ITD and FLT3/D835Y), not in FLT3 wild type (wt). The authors presumed that EGCG functions as an Hsp90 inhibitor and interferes with its chaperone activity for a client protein. Thus, dissociation from Hsp90 chaperone protein makes FLT3 mutant protein unstable and subject to degradation. FLT3 as the client protein of Hsp90 will be discussed in the next section.

### 2.3. FLT3 as Hsp90 Client Protein

Several papers have been published regarding the FLT3 protein as an Hsp90 client. Hsp90 inhibitors such as herbimycin A, geldanamycin, 17-AAG, and 17-dimethylaminoethylamino-17-demethoxygeldanamycin (17-DMAG) were employed to investigate the client protein of Hsp90. The first clue suggesting the FLT3 protein as anHsp90 client was from the study of myeloid cell line 32D transformed with FLT3-ITD, termed TDFLT3/32D in Minami et al.’s paper [20]. Treatment of herbimycin A resulted in the dissociation of FLT3-ITD from Hsp90 protein, as demonstrated by immunoprecipitation. Interaction between FLT-wt and Hsp90, however, was not observed. These results suggest that only FLT3-ITD, not FLT-wt, is the client of Hsp90 protein.

A report by Yao et al. supports the role of Hsp90 as a chaperone for FLT3 protein using 17-AAG as an Hsp90 inhibitor in AML cell lines [18]. Treatment of 17-AAG reduced the expression of FLT3 and disrupted the binding with Hsp90. The big difference between this study and previous research [20] is that both FLT3-wt and FLT3-ITD were affected by 17-AAG treatment. These data indicate that FLT3-wt, as well as FLT3-ITD, are client proteins of an Hsp90 chaperone. Follow-up research projects by the same research group investigating the combination treatment of 17-AAG with other anticancer agents confirmed 17-AAG induced degradation of FLT3-wt and FLT3-ITD [21,22]. 

A paper by Al Shaer et al. used 17-AAG as an Hsp90 inhibitor in primary AML cells expressing FLT3-ITD or FLT3-wt [23]. The 17-AAG treatment induced dissociation of FLT3-ITD from Hsp90, thereby reducing FLT3-ITD protein expression. Association between FLT-wt and Hsp90 was not observed from immunoprecipitation experiments.

Oshikawa et al.’s publication confirmed the effect of 17-AAG on the FLT3 degradation [24]. MV4;11 cells harboring the FLT3-ITD mutation and murine IL-3-dependent 32Dcl3 cells with inducible expression of FLT3-wt or FLT3-ITD were employed in this study. Treatment of 17-AAG induced the decline of expression of both FLT3-wt and FLT3-ITD. The sensitivity to 17-AAG was higher in FLT3-ITD than FLT3-wt. Furthermore, the authors reported that E3 ubiquitin ligases c-Cbl and Cbl-b mediated the proteasomal degradation of FLT3 by 17-AAG induction, and the phosphorylated form of FLT3 is more susceptible to 17-AAG-induced degradation.

The following paper by Ly et al. investigated FLT3 protein fused with ETS-translocation variant 6 (ETV6 or TEL) called ETV6/FLT3 fusion protein [25]. This research was performed by the same group who reported the green tea catechins as an Hsp90 inhibitor inducing FLT3 degradation as described above [19]. ETV6/FLT3 is an oncoprotein found in myeloid/lymphoid neoplasms. ETV6/FLT3 fusion protein was associated with Hsp90 protein in cos7, 293FT, and HeLa cells transiently transfected with plasmids expressing ETV6/FLT3 [23]. Treatment of 17-AAG in these cell lines transiently transfected with ETV6/FLT3 resulted in the reduction of ETV6/FLT3 expression.

The effects of various Hsp90 inhibitors (geldanamycin, 17-AAG, and 17-DMAG) were compared in 32D cells expressing FLT3 mutants [26]. FLT3 mutants and double mutants such as ITD, D835Y, ITD/N676K, ITD/F691L, ITD/F691I, ITD/G697R, and ITD/A848P are also compared for sensitivity by Hsp90 inhibitor treatment. Hsp90 inhibitors induced cytotoxicity in cells expressing FLT3 mutants. These results suggest that Hsp90 inhibitors can overcome drug resistance caused by FLT3 mutations. 

Based on the above publications using Hsp90 inhibitors and co-immunoprecipitation of Hsp90 and FLT3, FLT3-ITD proteins can be concluded as clients of Hsp90. However, data are inconsistent if FLT-wt is a client of Hsp90 or not. Four papers described that FLT3-wt is affected by 17-AAG in [18,21,22,24], and two papers reported a lack of interaction between FLT3-wt and Hsp90 [20,23]. Further research with elaborate design will be needed to resolve these discrepancies. Following the term ‘chaperone addiction’ proposed by Paul Workman, selective targeting of FLT3-ITD as an Hsp90 client protein can be employed for cancer therapy [27]. 

## 3. Proteasome Inhibitor

Bortezomib is an anticancer agent currently used in the clinic for multiple myeloma via the mechanism of proteasome inhibition [4]. Treatment of bortezomib to AML cells expressing FLT3-ITD, as well as FLT-wt, resulted in the degradation of FLT3 protein [28]. Along with FLT3 degradation, bortezomib also induced AML cell cytotoxicity. Interestingly, bortezomib induced FLT3-ITD degradation through autophagy in leukemic cells, as demonstrated by the treatment with a pharmacological inhibitor of autophagy. With the treatment of bortezomib, FLT3-ITD molecules were detected in the autophagosome. As a result, bortezomib overcame acquired resistance to quizartinib, a FLT3 inhibitor. Altogether, this report shows that proteasome inhibitors use the autophagy mechanism to degrade FLT3.

## 4. Arsenic Trioxide

Arsenic trioxide (ATO) is a currently used drug indicated for acute promyelocytic leukemia, a subtype of AML harboring PML-RARα fusion, often in combination with all-trans retinoic acid (ATRA) [4]. Treatment of ATO to AML cell lines with FLT3-ITD or FLT3-wt reduced the expression of FLT3, and this effect is enhanced with cotreatment of ATRA [29,30]. Consequently, ATO and ATRA exhibited cytotoxicity to AML cell lines. 

Nagai et al.’s report investigated the mechanism of ATO-induced FLT3 degradation [31]. Treatment of ATO resulted in the ubiquitination of FLT3-ITD, leading to its degradation via the ubiquitin-proteasome pathway. Weak ubiquitination of FLT3-wt is observed compared with FLT3-ITD, indicating the effects of ATO are selective to FLT3-ITD. The ubiquitination system of FLT3 protein will be discussed in the separate section below.

## 5. HDAC Inhibitors

A variety of histone deacetylase (HDAC) inhibitors can contribute to antileukemic activity by increasing the acetylation of histone or nonhistone proteins [32]. LAQ824 is an HDAC inhibitor with a cinnamyl hydroxamate structure and exerts antileukemic activity against the AML cell line MV4-11 and RS4-11 [33]. Further experiments revealed that treatment of LAQ284 reduced the expression of FLT3 protein in AML cell lines. The mechanism of LAQ284’s effect is presumed to be acetylation of Hsp90, thereby inhibiting the chaperone function of Hsp90. Dissociation of Hsp90 and FLT3 caused by Hsp90 acetylation results in polyubiquitination and proteasomal degradation of FLT3. Follow-up research by the same group confirmed the effects of HDAC inhibitors, using another small molecule HDAC inhibitor, LBH589 [34]. Furthermore, LBH589 treatment induced the acetylation of Hsp90 and increased the polyubiquitination of FLT3. Consistent with these observations, a combination treatment of LBH589 with 17-AAG exhibited synergistic effects on the FLT3 degradation and apoptosis of AML cell lines.

## 6. RET Inhibitors

Functional genomic studies were performed to identify the essential genes for AML cell viability, and proliferation [35]. The RET gene, encoding the receptor tyrosine kinase, was identified as an essential gene in AML cell survival. Authors found out that RET stabilizes FLT3-ITD proteins through inhibition of autophagy, and downregulation of RET expression using RET shRNA-induced autophagic degradation of FLT3-ITD. Inhibition of RET activity by specific RET inhibitors (vandetanib and danusertib) also resulted in autophagy induction and subsequent FLT3 degradation. Although a precise mechanism is not yet elucidated, loss of RET activity causes FLT3 downregulation, presenting RET as a therapeutic target for FLT3-dependent AML.

## 7. FLT3 PROTAC

PROTACs are bifunctional compounds designed to degrade target protein by guiding specific protein to the ubiquitin-proteasome system [1]. The multi-kinase degrader TL12-186 is designed by conjugating two moieties, ligand for the ATP-binding site of kinases and E3 ubiquitin ligase [36]. One of the kinases affected by TL12-186 is the FLT3 protein. TL12-186 treatment reduced the expression of FLT3 and inhibited the proliferation of AML cells lines harboring FLT3-ITD. This report presented a proof-of-concept study for PROTAC for FLT3 protein, and other FLT3 PROTACs are being reported on [37,38]. A recently reported global map of kinase degradability will provide a useful rationale for developing FLT3 PROTAC as an anti-leukemic agent [39]. 

## 8. Ubiquitin-Proteasome System for FLT3

Hsp90 inhibitors, arsenic trioxide, and HDAC inhibitors all induce degradation of FLT3 via the mechanism of the ubiquitin-proteasome system (UPS). Polyubiquitination is carried out by ubiquitin-activating enzymes (E1s), ubiquitin conjugases (E2s), and ubiquitin ligases (E3s) [40]. Ubiquitin ligases confer specificity for the substrates to be ubiquitinated. Polyubiquitinated proteins linked at lysine 48 of ubiquitin are subjected to degradation by the proteasome [40]. Several E3 ubiquitin ligases are reported to contribute to the ubiquitination of FLT3 protein, and c-Cbl and Cbl-b are reported as E3 ligases for FLT3 [24,41]. E3 ubiquitin ligase SIAH1, in concert with E2 ubiquitin conjugase UBCH8, also contributes to the polyubiquitination of FLT3 [42].

Another component of UPS is deubiquitinases (DUBs), removing ubiquitin from ubiquitinated substrates [43]. The DUB for FLT3-ITD protein, USP10, is identified from the DUB-inhibitor library screen [44]. Consequently, inhibition of USP10 by small molecules reduced the expression of FLT3-ITD, but not FLT3-wt, along with cytotoxicity to AML cells [44]. The discovery of novel USP10 inhibitors is followed. Wu-5, an USP10 inhibitor with IC_50_ 8.3 μM in DUB-labeling assay, induced FLT3 degradation in AML cell lines harboring FLT3 [45]. FLT3 reduction by Wu-5 treatment is observed only in cells with FLT3-ITD, not FLT3-wt.

## 9. FLT3 Phosphorylation Status and Degradation

The status of FLT3 phosphorylation and activation appears to be related to the susceptibility of FLT3 degradation. From the reports of small molecule-induced FLT3 degradation described above, a few clues can be found regarding the difference in the degree of FLT3 degradation, between the phosphorylated and the unphosphorylated form of FLT3. The majority of papers demonstrated that FLT3-ITD is more susceptible than FLT3-wt to small molecules’ degraders [19,20,23,29]. These observations suggest that the characteristics of FLT3-ITD as an autophosphorylated and activated form can affect the protein degradation process, such as UPS. 

Oshikawa et al.’s paper., reporting 17-AAG-induced degradation and involvement of Cbl ubiquitin ligase as described above, examined the effect of the tyrosine kinase inhibitor sorafenib on the 17-AAG sensitivity [24]. Pretreatment with sorafenib reduced the degree of degradation by 17-AAG, implying that the inactive state of FLT3 induced by sorafenib can protect from the degradation process.

In the report investigating ATO-induced FLT3 downregulation, sorafenib’s effects on ATO-induced phenomenon were examined [31]. Sorafenib treatment to AML cell lines increased FLT3 expression, partially via transcriptional activation. In addition to increased transcription, reduced degradation of FLT3 also contributed to upregulation of FLT3 expression, as shown by reduced FLT3 ubiquitination upon sorafenib treatment. In the presence of sorafenib resulting in an inactive state of FLT3, AML cells became less sensitive to ATO-induced degradation and ubiquitination. 

Although only one small molecule (sorafenib) was tested in the papers above [24,31], the phosphorylation status appears to be important in determining the fate of FLT3 toward the degradation process. Research regarding the relationship of FLT3 conformation and the degradation process can provide a clear view of these phenomena. In addition, more specific small molecule FLT3 inhibitors, such as quizartinib and gilteritinib, need to be used to exclude off-target effects of sorafenib.

## 10. Conclusions

**Figure 1:**
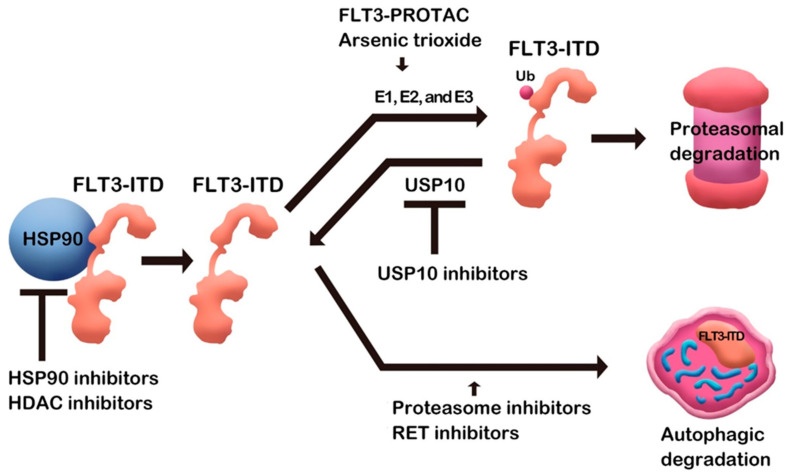
FLT3 degradation by small molecules.

Quite a few small molecules induced FLT3 degradation (Table 1). Some small molecules binding directly to FLT3 protein may have specificity to FLT3, while compounds such as Hsp90 inhibitors can be nonspecific, affecting many Hsp90 clients. As a mechanism of degradation, some compounds induce proteasomal degradation, and autophagic degradation is also employed in FLT3 downregulation (Figure 1). Although various mechanisms are involved, as described above, target protein degradation, at least partially, contributes to the antileukemic effects in addition to kinase activity inhibition. Therefore, the research compiled above will open an avenue for FLT3 degraders as AML therapeutic agents. After completing the FLT3 degrader study in AML, it will be extended to all treatments.

## Figures and Tables

**Table 1:** Small molecules inducing FLT3 degradation.

FLT3 Degrader	Mechanism	Reference
17-AAG	Hsp90 inhibition	[18]
EGCG, EGC, ECG	Hsp90 inhibition	[19]
Bortezomib	Proteasome inhibition	[28]
Arsenic trioxide	FLT3-ITD ubiquitination	[29,30]
LAQ284	HDAC inhibition	[33]
LBH589	HDAC inhibition	[34]
Vandetanib	RET inhibition	[35]
Danusertib	RET inhibition	[35]
Wu-5	USP10 inhibition	[45]
FLT3-PROTAC	PROTAC	[36,37,38]

17-AAG, 17-allylamino-17-demethoxy-geldanamycin; EGCG, epigallocatechin gallate; EGC, epicatechin gallate; ECG, epicatechin-3-gallate; Hsp90, heat shock protein 90; HDAC, histone deacetylase; USP10, ubiquitin-specific protease 10; PROTAC, proteolysis-targeting chimera.

## Data Availability

Data sharing not applicable.
